# Seroprevalence of Pertussis in Senegal: A Prospective Study

**DOI:** 10.1371/journal.pone.0048684

**Published:** 2012-10-31

**Authors:** Lobna Gaayeb, Jean Biram Sarr, Mamadou O. Ndiath, Jean-Baptiste Hanon, Anne-Sophie Debrie, Modou Seck, Anne-Marie Schacht, Franck Remoué, Emmanuel Hermann, Gilles Riveau

**Affiliations:** 1 Centre for Infection and Immunity of Lille (CIIL) - U1019 Inserm, UMR8204 CNRS, Institut Pasteur de Lille, Lille, France; 2 Biomedical Research Centre EPLS, Saint-Louis, Senegal; 3 Université Lille Nord de France, Lille, France; 4 Laboratoire de Paludologie, UMR198 URMITE, IRD Campus international de Hann, Dakar, Senegal; 5 UMR MIVEGEC – IRD224-CNRS5290-Université de Montpellier 1 et 2- IRD – Centre de Recherche Entomologique de Cotonou (CREC), Cotonou, Benin; Melbourne School of Population Health, Australia

## Abstract

**Background:**

Pertussis, also known as whooping cough, is a vaccine-preventable respiratory disease caused by ***Bordetella pertussis*** infection, against which Senegalese children are immunized with the diphtheria-tetanus-whole cell pertussis vaccine (***DTwP***). Seroepidemiology of pertussis has been widely described in industrialized countries, but rare are the studies referring to it in developing countries.

**Methods:**

We conducted a longitudinal survey in Northern Senegal to investigate the epidemiology of ***B. pertussis*** by evaluating the IgG antibody (Ab) response against pertussis toxin (PT). A cohort of 410 children aged 1 to 9 from five villages in the Middle Senegal River Valley were followed-up for 18 months. During that period, five visits were made to assess the immunological status of the children.

**Principal Findings:**

PT-specific IgG responses were significantly different according to age. Until the age of 3, there was a decrease in the Ab response, which then increased in the older groups. Assessment of IgG antibodies to PT (IgG-PT) suggested evidence of recent exposures to the pathogen. Surprisingly, in one of the five villages the average Ab response to PT was very low at all ages during the first 6 months of the study. At the third visit, IgG-PT concentrations peaked to very high levels, to slightly decline at the end of the survey. This indicates an outbreak of ***B. pertussis***, whereas in the other villages a pertussis endemic profile could be observed.

**Conclusions:**

Pertussis is endemic in Northern Senegal despite the introduction of vaccination. The circulation of the bacteria seems to differ between geographic locations and over time. A more complete understanding of the epidemiology of pertussis and its environmental determinants could provide information to adapt vaccination programs.

## Introduction

Whooping cough (or pertussis) is a highly infectious respiratory disease caused by the bacterium *Bordetella pertussis*, and is transmitted from infected to susceptible individuals through droplets.

The World Health Organization (WHO) estimates that in 2008 about 16 million cases of pertussis occurred worldwide, 95% of which were in developing countries, causing the death of 195 000 children. Since the widespread introduction of the whole cell pertussis vaccine, a partial herd immunity was achieved resulting in a significant drop in the incidence of pertussis [1]–[2]. Mean duration of vaccine-induced immunity is estimated to range from 5 to 15 years [3]–[4]. However, since mid- eighties, a slow but constant increase in pertussis incidence has been reported in many industrialized countries, despite their high vaccination coverage, and the disease continues to be a public health concern [5].

Several reasons have been suggested to explain this resurgence: an increased awareness of the disease and a recognition of atypical forms in adolescents and adults [6], a possible adaptation of the pathogen consequent to vaccine selective pressure [7], and waning of vaccine-induced immunity, which leads to the occurrence of pertussis infection in previously vaccinated individuals [4].

Various studies have been undertaken describing the epidemiology of pertussis in industrialized countries, but seldom have they been carried out in developing countries [8], [9]. In Africa, a major study has been conducted from 1990 to 1994 in the well-monitored region of Niakhar: the Senegal pertussis trial, to compare the efficacy of a cellular and an acellular pertussis vaccine [10]–[11]. Samples collected during this trial have been used to establish a link between serological and clinical diagnosis of the disease [12], while other studies have described the epidemiology of the disease since the introduction of vaccination [13]–[14]. In sub-Saharan settings, where reporting of pertussis is restricted to the classic clinical picture observed in toddlers, and disease reporting is exclusively passive, seroepidemiology seems to be a highly valuable tool to assess the prevalence of pertussis infection in the area.

Our present study was located in Northern Senegal, where we investigated the seroepidemiology of *B. pertussis* by evaluating the antibody response to pertussis toxin (PT). Analyses were performed on sera of more than 400 children from five villages of Podor district that we followed up for one year and a half. Results from our study strongly support the circulation of *B. pertussis* and the presence of endemic villages as well as outbreak episodes of *B. pertussis* in the area.

## Materials and Methods

### Study Area and Population

In this longitudinal follow-up study that took place between October 2008 and January 2010, 410 children aged 1 to 9 years were followed in five study sites from the Northern region of Senegal, belonging to the Podor district (Saint Louis Region). Five villages were concerned by this study: “Agniam Towguel” (16°32′ N-14°48′ W; total population (TP): 989; several temporary ponds, traditional housing, irrigated crops), “Fanaye Diery” (16°32′ N-15°13′ W; TP: 6781; animal husbandry, irrigated crops, some urbanized habitat), “Niandane” (16°35′ N-14°59′ W; TP: 5100; Rice and banana farming, irrigated crops, some urbanized habitat), “Ndiayène Pendao” (16°30′ N- 15°03′ W; TP: 2734; animal husbandry, Savannah surroundings), “Guédé Village” (16°33′ N-14°48′ W; TP:3005; Traditional housing, irrigated crops). In this region, the climate is Sahelian, with annual rainfall between July and September (340 mm during 2009). Mean temperature ranges between 20°C and 30°C during the cool season (November to February) and 25°C to 38°C during the warm season (March to October).

**Figure 1:**
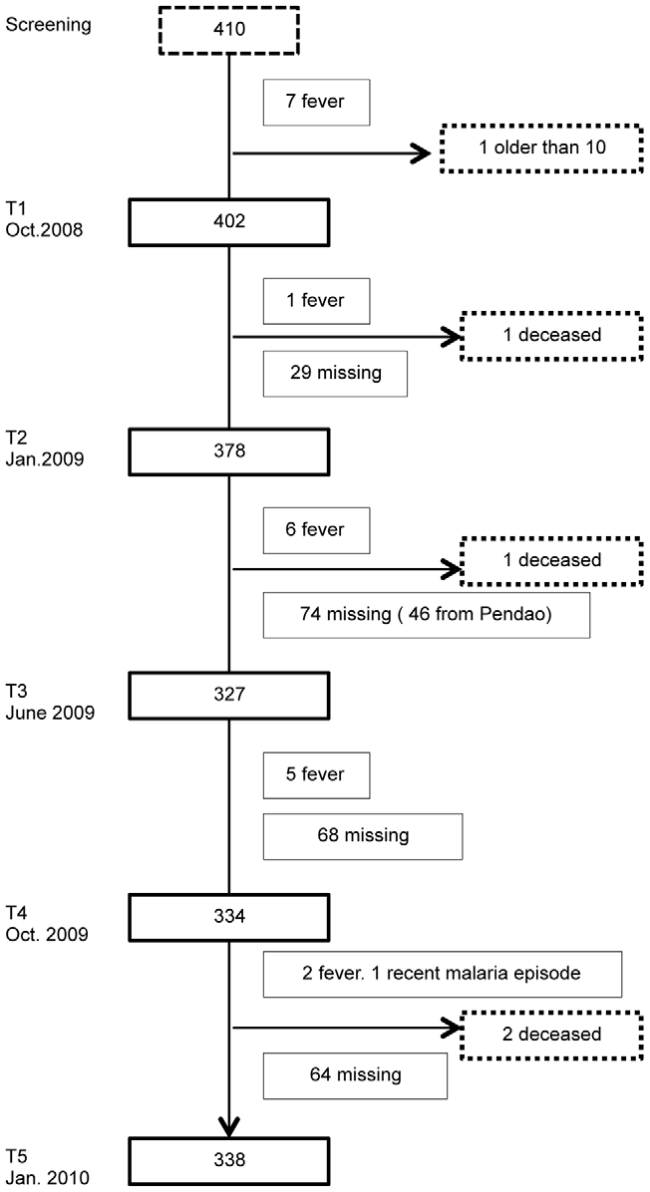
Flow of participants through the study.

The number of children recruited in each village was proportional to the total population of the village. Agniam and Guédé are more rural and less populated compared to the other 3 villages of the study (Fanaye, Niandane and Pendao). Villages were visited five times during the survey, at T1 (October 2008), T2 (January 2009), T3 (June 2009), T4 (October 2009) and T5 (January 2010). A flowchart of the study is presented in figure 1.

The WHO Expanded Program on Immunization (EPI) started in 1986 in Senegal, and recommends a schedule of diphtheria-tetanus-whole pertussis vaccine (DTwP) vaccination at 6, 10 and 14 weeks of age. The immunization coverage is estimated to be 86% in the Saint-Louis Region (personal communication of Mrs. Diaw, head of EPI program for St-Louis Region). Since 2005, Quinvaxem ® (Crucell), a pentavalent whole-cell pertussis vaccine, has been used, and before that, it was the trivalent whole cell pertussis vaccine from Pasteur-Mérieux which was administered to the children. No additional vaccine was administered to the children of the cohort during the study.

**Table 1:** Description of the cohort at the beginning of the survey (T1).

	Total	Agniam	Fanaye	Niandane	Pendao	Guédé	*p*
n	402	52	103	103	98	46	
Age (years) (Median and quartiles)	5.2 (2.6; 7.5)	3.7 (2.6; 6.5)	6.3 (2.8; 7.3)	5.6 (3.0; 7.5)	3.9 (2.1; 7.0)	7.1 (2.7; 8.6)	***0.0278*** a
Gender (%)	*0.48* b						
Boys	200 (49.8)	26 (50)	48 (46.6)	58 (56.3)	49 (50)	19 (41.3)	
Girls	202 (50.2)	26 (50)	55 (53.4)	45 (43.7)	49 (50)	27 (58.7)	
DTwP1 vaccination status b							
Vaccinated (%)	230 (57.2)	51 (98.0)	74 (71.8)	39 (37.9)	61 (62.2)	5 (10.9)	***<0.0001*** b
Age	3.1 (1.9; 5.5)	3.7 (2.6; 6.5)	4.7 (2.1; 6.8)	2.7 (1.7; 3.4)	2.4 (1.7; 3.7)	2.2 (1.7; 3.7)	***<0.0001*** a
Unknown status (%)	170 (42.3)	1 (2.0)	28 (27.2)	64 (62.1)	36 (36.7)	41(89.1)	
Age	7.1 (6.0; 8.0)	8.0	7.3 (6.7; 7.9)	6.8 (5.8; 7.7)	7.2 (6.6; 8.2)	7.6 (2.9; 8.9)	

a ANOVA test of age differences between children of all villages, and between ages of children of all villages according to their vaccination status.

b Chi-square test to assess the gender distribution among villages and distribution of children within villages according to their vaccination status.

2 children were not vaccinated, one from Fanaye, and the other from Pendao.

### Ethics Statement

The project was approved by the National Ethics Committee of Senegal (Approval Number: SEN26/08) (Clinicaltrials.gov ID: NCT01545115). Written individual informed consent was obtained from each participant's parent or legal guardian at the beginning of the survey, and at each visit child's and parent's approval was sought orally, in accordance with the Declaration of Helsinki.

**Table 2:** PT-IgG responses at T1.

	Total	Agniam	Fanaye	Niandane	Pendao	Guédé	*p*
n	402	52	103	103	98	46	
Anti-PT IgG (IU/ml) (median,quartiles)	14.5 (6.6; 47.3)	11.7 (6.6; 28.3)	18.9 (7.6; 85.0)	12.8 (7.0; 41.5)	22.9 (4.9; 64.7)	8.5 (5.0; 16.9)	*0.2078* a
IgG >30IU/ml; n (%)	135 (33.6)	11 (21.2)	44 (42.7)	32 (31.1)	43 (43.9)	5 (10.9)	***0.0001*** b
IgG >80IU/ml; n (%)	65 (16.2)	3 (5.8)	27 (26.2)	11 (10.7)	23 (23.5)	1 (2.2)	***<0.0001*** b

a One-way ANOVA between IgG responses according to villages.

b Chi-square test between seroprevalence according to villages.

**Table 3:** PT-IgG >30 IU/ml at T1 according to age categories and villages.

Age category	All villages	Agniam	Fanaye	Niandane	Pendao	Guédé
1–2 years	45% (65)	50% (6)	56% (18)	36% (14)	45% (22)	20% (5)
2–3 years	34% (62)	40% (15)	44% (9)	33% (12)	31% (16)	20% (10)
3–4 years	19% (42)	13% (8)	33% (6)	21% (14)	17% (12)	0% (2)
4–5 years	26% (27)	0% (6)	56% (9)	33% (3)	20% (5)	0% (4)
5–6 years	32% (28)	0% (3)	40% (5)	29% (14)	50% (6)	(0)
6–7 years	33% (58)	0% (2)	39% (23)	17% (18)	50% (14)	0% (1)
7–8 years	33% (57)	20% (5)	29% (21)	47% (15)	50% (10)	0% (6)
8–9 years	37% (63)	0% (7)	50% (12)	38% (13)	77% (13)	11% (18)

Percentages of children with PT-IgG >30 IU/ml at the first visit in every village.

Total number of children within age category is in brackets.

**Table 4:** Anti-PT seroprevalence at T1 in all villages according to age category.

		Total	Agniam	Fanaye	Niandane	Pendao	Guédé	*p*
N	≤3 years	127	21	27	26	38	15	*0.1107* a
>3 years		275	31	76	77	60	31	
Seropositives (IgG >30IU/ml) (%)	≤3 years	39.4	42.9	51.9	34.6	39.5	20.0	*0.3457* a
> 3 years		30.9	6.5	39.5	29.9	46.7	6.5	***<0.0001*** a
*p* a		*0.1117*	***0.0039***	*0.3652*	*0.6343*	*0.3114*	*0.5352*	
Anti-PT IgG (IU/ml) (median, quartiles)	≤3 years	19.5 (7.3; 60.0)	28.8 (11.6; 54.0)	37.0 (9.6; 96.5)	15.3 (8.3; 42.8)	18.0 (1.2; 56.5)	8.0 (3.3; 26.4)	*0.1055* b
>3 years		13.0 (6.3; 44.5)	9.4 (5.6; 19.2)	16.1 (7.3; 76.7)	12.6 (6.7; 43.0)	26.7 (5.0; 89.0)	10.17 (5.0; 16.8)	***0.0043*** b
*P* c		*0.2143*	***0.0003***	*0.4245*	*0.5820*	*0.3916*	*0.6393*	

a Chi-square test has been performed.

b Kruskal-Wallis test between IgG responses according to the villages.

c Mann-Whitney test has been performed for children of the same villages.

### Data collection

At each visit, a questionnaire was filled in for every child. We reported the content of available vaccination documents to assess date of birth, uptake and timeliness of vaccination. Vaccination history was collected on oral communication basis for those who have lost their vaccination cards. For schoolchildren, date of birth was ascertained from school register.

**Figure 2:**
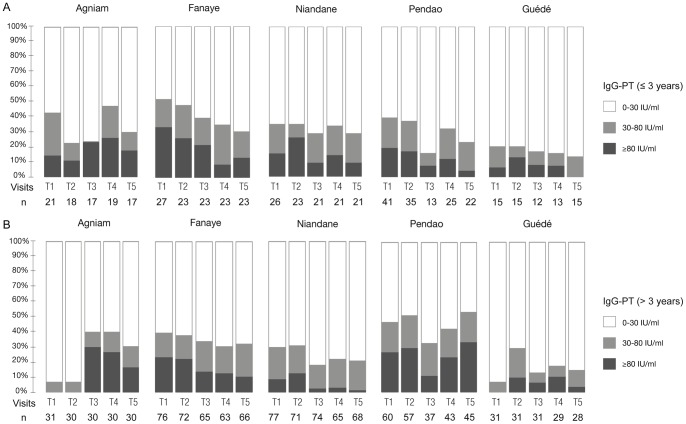
Seroprevalence of anti-PT IgG concentrations according to the village and visit for children younger (panel A) and older than 3 years (panel B). Chi-square test between seropositive children (anti-PT IgG concentration >30 IU/ml) and seronegative children at each visit within villages: panel A.: Agniam, **p = 0.3588**; Fanaye, p = 0.5227; Niandane, p = 0.4056; Pendao, p = 0.4276; Guédé, p = 0.9928; panel B.: Agniam, **p<0.0008**; Fanaye, p = 0.7641; Niandane, p = 0.2280; Pendao, p = 0.3292; Guédé, p = 0.1754.

### Blood collection and serology

Body temperature was measured by means of auricular thermometer at all visits. Data from children having over 38.5°C body temperature were not used. The children who were unwell were not included. Blood was collected by finger prick on BD Microtainer® tubes (Beckton Dickinson, USA) and centrifuged for 10 min at 3000 rpm. Sera were kept at −20°C as 0.15 ml aliquots in 96-well 0.8 mL deep-well storage plates (ABgene, Thermo Scientific, USA) until analysis. Serum antibody concentrations were analyzed by enzyme-linked immunosorbent assay (ELISA).

**Figure 3:**
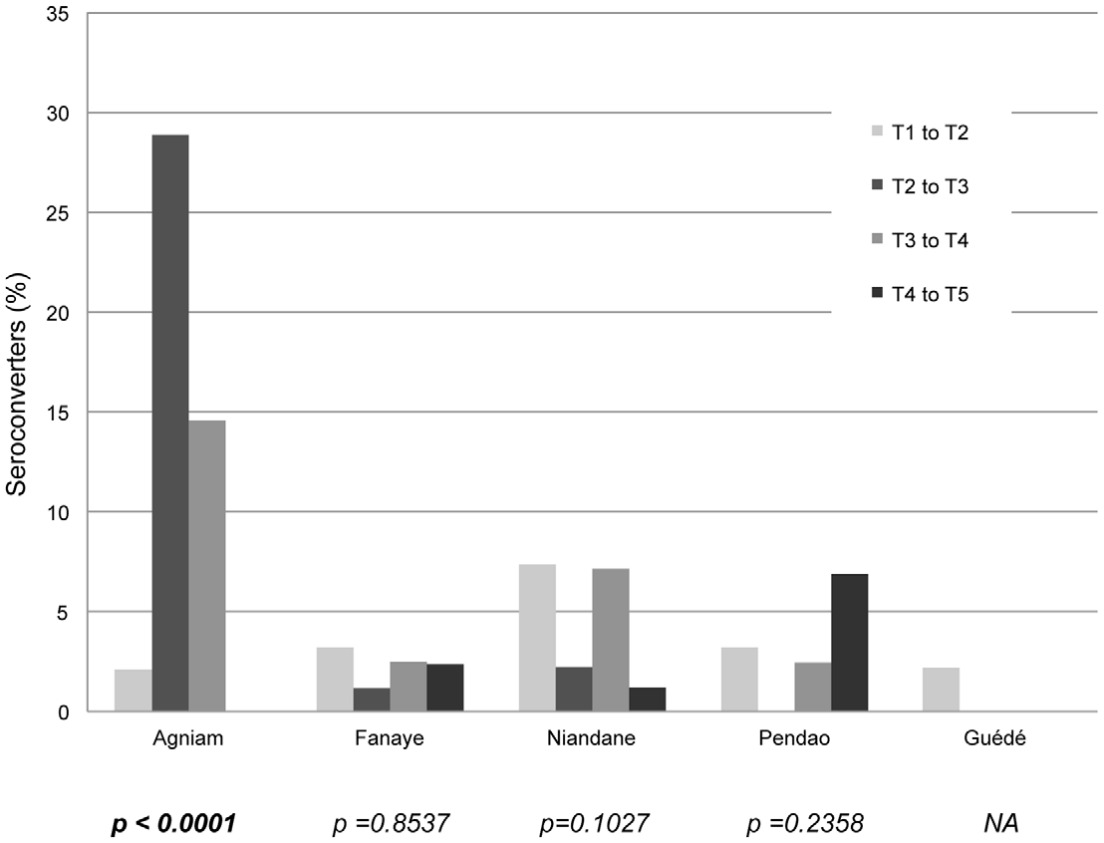
Seroconversion throughout the visits in each village. Chi-square test between number of seroconverters (sera becoming newly positive to PT) and rest of population from the village.

IgG antibodies directed against *B. pertussis* toxin (PT) were measured using a commercial kit: *Bordetella pertussis* toxin IgG (ESR1201G, Virion\Serion GmbH, Germany) on a 1∶100 diluted sera, following the kit recommendations. Samples with very high antibody concentrations (rated >max by the software provided along with the kit) were retested at a higher dilution (1∶500) so that the concentrations would fall within the optimal detection ranges. Serum values for *B. pertussis* were assigned in IU/ml according to the “Serion Activity Quantification Software v8.1”, which allowed transformation of OD units into IU/ml according to the OD of a standard sample run on the same plate, using a 4-parameter Logistic Model [15]. It also allowed rating of samples as negative (<20 IU/ml), borderline (between 20 and 30 IU/ml), or positive (>30 IU/ml). During the analysis we have considered samples with <30 IU/ml negative and samples > or  =  to 30 IU/ml as positive. Moreover, anti-PT IgG concentrations >80 IU/ml detected in a serum sample were considered as an indicator of a recent *B.pertussis* infection or immunization [16]. We have chosen a dual cut-off value, to ensure serodiagnostic sensitivity (lower value) as well as specificity (higher value). All ELISA plates were run on a semi-automated washer (Skatron Skanwasher 400, Molecular devices, UK) and read on a Multiskan MS reader (Thermo Labsystems, USA). The results were expressed in units of OD by the microplate analysis software Deltasoft (Biometallics, USA).

### Data Management and Statistics

Data were entered by two persons into a Microsoft Access Database and crosschecked by the data manager. Chi-square test was used to determine the heterogeneity between distributions. The non-parametric Mann-Whitney test was carried out to compare the distributions of 2 independent groups. Differences between distributions of 3 or more samples were assessed using one-way analysis of variance (ANOVA) or the Kruskal-Wallis non-parametric test when the data did not follow a Gaussian distribution. The minimal level of significance was considered as *p*<0.05. Analyses were performed using Microsoft Excel 2007 and GraphPad Prism 5.04 (GraphPad Software, San Diego California, USA).

## Results

In this study, antibody responses to pertussis of children aged 1 to 9 years living in five villages of Podor district in Northern Senegal were analyzed. We focused our analysis on the IgG response to pertussis toxin, as the development of a humoral response to this antigen is known to be strictly correlated with natural infections to *Bordetella pertussis* [17]. Among the 410 children who were screened for this study, 402 children were included at the first visit (T1). An overview of the study population during the survey is shown in figure 1. Age of included children at T1 differed between villages, but there was no difference in gender distribution (Table 1).

### Immunization history of children

Table 1 shows information collected at T1 about the vaccination status of children for the first DTwP dose. Vaccination status was ascertained based on vaccination cards available for review or oral communication of the child's mother (228 (56.7%) and 7 (1.7%) respectively in total cohort). Two children who brought their card were not vaccinated. Children bearing a vaccination card were younger (median age 3 years) than those who did not have one (median age 7 years) (Mann-Whitney *p*<0.0001). Indeed, the individual vaccination status of the participants was known for 86.7% of the children younger than 5 (and 89% of those younger than 3), while only 29.6% of the children older than 5 brought their vaccination card, since the latter tended to be lost or deteriorated with time. Although most children were likely to have received three doses of diphtheria, tetanus, and whole-cell pertussis vaccine within the first year of life, those who did not bring their card and did not know whether they received the first DTwP, were referred to in table 1 as children with unknown vaccination status. Distribution of vaccinated and unknown vaccination status children was not the same across villages (p<0.0001). The different age pattern between villages likely accounts for this contrasting observation (Table 1).

### IgG responses to pertussis toxin vary with age

As measured at T1, PT-IgG concentrations did not differ between boys and girls from any village (data not shown). Given that differences in the trends of response to PT antigen were not notable between vaccinated children in comparison to those with an unknown vaccination status (data not shown), we pooled both groups. 33.6% of the global cohort presented anti-PT IgG concentrations higher than 30 IU/ml, and 16.2% higher than 80 IU/ml. However, seroprevalence rates were significantly different according to villages (table 2). When we compared the age-specific prevalence or levels of IgG directed to PT, we observed an inflexion point at around the age of three in the antibody response to PT in children of all villages (table 3). Before the age of three, anti-PT IgG concentrations decreased with age (children aged 2 to 3 compared to those aged less than 2). Once the children reached 4 to 5 years of age IgG concentrations were in average less age-dependent. PT seroprevalence remained steady at around 30% for the children older than 3, while it was 53% in the children aged 1 year (not shown). The inflexion point observed in children around the age of 3 indicated an antibody concentration decay until that age, and led us to compare the IgG response to pertussis between children belonging to different age categories: younger and older than 3 (table 4).

Indeed, absolute IgG values to PT were not statistically different between the two age categories: 19.5 (7.3; 60) for the children younger than 3 and 13 (6.3; 44.5) for those older than 3; Mann-Whitney *p = 0.2143*). The proportions of PT-seropositive and PT-seronegative children were similar between children younger and older than 3 (table 4, *p = 0.117*). Moreover, anti-PT IgG responses of 80 IU/ml or higher were detected in sera of children of all ages (Supplementary Material S1). Such antibody levels could be linked to a recent vaccination or contact with the bacteria [16] [18].

### IgG responses to pertussis antigens vary through villages

We next focused our analysis on the level of antibody response to pertussis toxin in both age categories according to the village of residence during the first visit.

The percentage of seropositive children was significantly different between villages, while the concentration of anti-PT IgG was not (Table 2). The proportion of PT-seropositive children ranged from 10.9% and 21.2% in the smaller villages of Guédé and Agniam, respectively, to a higher prevalence in the three larger villages of Fanaye, Niandane and Pendao. In addition, differences in seroprevalence rates through villages were only observed for the children older than 3 years (table 4, p<0.0001), while children younger than 3 had similar seroprevalence rates (table 4, p = 0.3457). Furthermore, a difference between age groups of the same village was solely measured in Agniam, where response to PT of the children older than 3 was significantly lower than those under 3 (table 4, p = 0.0039), while other villages displayed maintenance in the proportion of children positive for antibodies directed against PT throughout age categories. It is also in small villages (Agniam and Guédé) the weakest antibody responses in the older group of children were measured.

These observations might reflect endemic natural exposures to environmental bacteria in all villages at this first visit, but not in Agniam and Guédé. The detection of higher pertussis antibody concentrations in the younger children from Agniam was likely a remnant of the immunization process that progressively declined with age due to the lack of a natural endemic booster by environmental bacteria.

### IgG responses to PT: Longitudinal follow-up

We next followed for more than 1 year the immune response to pertussis with a total of five time points of analysis (T1 to T5). Variations in individual responses to PT, for all children at each visit according to age at first visit, are presented for each village in the Supplementary Material S1.

In figure 2, we have plotted the proportions of responders in each age category according to the residence and timing of visit. We observed different dynamics in PT-responses across villages and among age groups. For the children younger than 3 (figure 2a), there was no statistical difference in the proportions of responders in each village according to the time of visits (p>0.1 for all five villages). As for the children older than 3 (figure 2b), proportions of responders in Fanaye, Niandane, Pendao and Guédé did not vary significantly over time, whereas they did in the case of the village of Agniam (p = 0.0008). Although not significantly, the prevalence of highly positive sera to PT (anti-PT IgG concentration ≥80 IU/ml) tended to decrease steadily over time in Fanaye, for children from both age categories. The harsh decrease observed at visit 3 in the village of Pendao is probably due to an artifact since only 50% of the children were present at T3 (figure 1). In contrast, in the village of Agniam, a dramatic increase in the number of responders between the second and third visits was observed, and only started to decline after the fourth visit. In figure 3, we have illustrated the number of children shifting from PT-seronegative (IgG concentration ≤30 IU/ml) to PT-seropositive status. While in all other villages the seroconversion rate looked rather low and constant over time, in Agniam the number of seroconverting children clearly peaked between visits T2 and T3 (28.9% of children who were seronegative for PT became seropositive) and was stable between visits T3 and T4 (14% new seropositive children between these visits). On the other hand, 30% of Agniam's children doubled their anti-PT IgG concentration between T2 and T3 and 44% of them did between T3 and T4 (data not shown). During the outbreak, individual antibody dynamics reveal a tendency to a stronger increase in older children's IgG-PT as compared to younger children (Supplementary Material S1). These observations strongly support an outbreak of pertussis in the village of Agniam, probably starting between January (visit 2) and June (visit 3) 2009.

## Discussion

Seroepidemiological studies are recognized as a valuable tool to assess the circulation of *Bordetella pertussis* in communities. Although many studies have described the seroprevalence of pertussis in industrialized countries, only few teams have reported its incidence in developing countries. The aim of our work was to describe the serological response to *B. pertussis* antigens in a children cohort of Northern Senegal over an extensive period of time. We carried out a longitudinal survey where we assessed children's IgG response to pertussis toxin over a year and a half. In this study, circulation of *B. pertussis* was clearly shown by serological measurement with a great variation in serological responses to PT antigens according to age and village of residence.

Infections with *B. pertussis* persist in vaccinated populations and these may remain unrecognized because of the atypical or mild nature of symptoms [19]. In our study, the absence of data on typical or atypical pertussis episodes or clinical evidence of exposure to pertussis in our cohort may be perceived as a critical point; however, accurately estimating the incidence of pertussis by means of clinical observations, especially in an African rural context seems challenging because of misdiagnosis and underreporting. Therefore, seroepidemiology has proven to be a valuable tool for the assessment of the circulation of the bacteria and the profile of the response to its antigens according to age, location and time of the year.

Pertussis toxin is one of the major virulence factors of *B. pertussis* and anti-PT antibodies are specific for *B. pertussis*. A single cut-off value is now acknowledged as a reliable indicator of a contact with the pathogen [16] [18] [17] [20]. However, high levels of IgG responses to PT antigen cannot discriminate between antibodies induced by vaccination or those due to infection. In the present work, IgG-PT concentrations higher than 80 IU/ml have been measured in all age categories, indicating a likely contact with *B. pertussis*, or in the case of younger children a recent vaccination. This cut-off value may not be generally valid, as studies have suggested values between 50 and 100 IU/ml as an indicator of recent infection, depending on the endemic situation, age groups, etc. [18]. However, in the absence of revaccination, the increase in antibodies between visits, which we observed in many children, indicate that they have been in recent contact with a circulating *B. pertussis.* Additionally, high levels of anti FHA antibodies as observed in our study (data not shown) might also reveal some contacts with *B. pertussis* as well as with other bacteria such as other Bordetella species [21] or other infectious agents including *Haemophilus inﬂuenzae* [22], *Mycoplasma pneumonia* and *Chlamydia pneumonia* [23] among others.

Our results show that waning of anti-PT IgG response is particularly remarkable as IgG responses to pertussis antigens tended to decrease with age until around 3 years. This trend is in accordance with previous studies in the Western countries, where it has been demonstrated that between 1 to 3 years after whole-cell vaccination, children's antibody levels were almost undetectable and children became probably more susceptible to pertussis at 4 years of age [4]. A seroprevalence below 50% for PT in children younger than three may also emphasize that schedule for pertussis immunization may not be adequate to reach a sufficient herd immunity level in our cohort. Therefore young populations could be at risk for pertussis outbreaks in the absence of a vaccine boost. However, long-term protein-specific memory B cells could persist and provide protection against pertussis [24]. Immunization has also been demonstrated to increase the mean age at infection (from 4.7 years in the pre-vaccination era to 6.3 years in the post vaccination era) in children who were residents of the Niakhar study area in Senegal [13] [25] [3]. This drop in incidence of pertussis transmission after vaccination of Senegalese children from Niakhar is consistent with important herd immunity that likely induced a protective effect against pertussis in Africa in spite of the rapid loss of immunity, as supported by our present study. However, the decrease of antibodies until the age of 3 could be concomitant to an emergence of mild or subclinical infection episodes in children upon greater exposure to the bacteria, which were not diagnosed by health-care providers in the present work. Indeed, the observation of IgG-PT increase in a number of children after the age of 3 to 4 years clearly indicates a recent boost after the onset of natural pertussis infection, because none of the children received any vaccine during our study period. The increased responses in children above 3–4 years to PT could be due to high human contact levels in this age group (household members, schooling, greater range of social interaction, etc.). In various outbreaks across Europe and the USA transmission between schoolmates was clearly demonstrated, emphasizing the role of older children in the transmission of pertussis to schoolmates [26], [27]. The impact of circulating bacteria in the maintenance of immune memory to pertussis and consequently in preventing transmission or clinical disease remains nonetheless to be studied in more detail in our setting. Further investigations, including a clinical follow-up of the children in parallel to serological measurements should be carried out, to investigate whether high antibody responses to PT could be correlated with any symptoms in this population.

Findings of our longitudinal study about individual seroconversion support the endemicity of *B. pertussis* in some villages whereas it seemed to have an epidemic profile in others despite high vaccination coverage. Also, in the epidemic setting, seroconversions seemed to happen earlier, and IgG-PT reached higher levels in older children. The tendency in the children younger than 3 to respond more slowly and in a lower magnitude to pertussis infections has already been described in industrialized countries [28]. This could be due to a faster clearance of the bacteria by the more recently immunized individuals, and/or may be related to the development, with increasing age, of a specific memory immunity through repeated encounters with *B. pertussis* antigens. Interestingly the villages where the bacteria may be in circulation are the most populated ones, whereas the serological responses were different in the less populated villages of Agniam and Guédé. In Agniam, a *B. pertussis* outbreak was witnessed as revealed by the high seroconversion rate at the end of the dry season, while in Guédé overall response was low, and seroconversion events rare, indicating a low-level circulation of *B. pertussis.* In the survey carried out in the Niakhar area, it has been reported that spatial pattern of whooping cough strongly depends on contact, which occurs more frequently in dense populations [29]. We hypothesize that in Agniam, a Critical Community Size below which the infection is not able to persist, was not reached, while in the other villages a sufficient level of contact enabled a continuous transmission of the bacteria and its circulation was not able to fade out. The outbreak of pertussis in the village of Agniam could be a result of an input of infected people from outside the community. In this particular context, presence of circulating *B. pertussis* is worthy of notice because it may lead to an overestimation of vaccination-induced antibodies persistence, especially in the youngest children, while these antibodies production may have been boosted by natural infections.

In summary, from a public health perspective, this work has revealed the endemicity of pertussis in the Northern area of Senegal. Locations, participants' number and age range, as well as the longitudinal follow-up are important assets of this study. Results of this work orientate towards the need to carefully monitor the epidemiology of vaccine-preventable diseases in a developing country context. This would support health authorities in the choice of the most adapted national vaccination program, which should be established in very close relation to the past and present epidemiological situation and available healthcare resources.

## Supporting Information

Supplementary Material S1 **Individual changes in anti-PT IgG responses over time according to age at first visit in all villages.** Different age groups are in separate panels; Data from each individual are connected. Red lines indicate median values at a given visit. The Kruskal-Wallis non-parametric test has been run for each age category in each village. Only significant test results are shown on the graph.
(TIF)
