# Supplementary figures and images for: Seroprevalence of Pertussis in Senegal: A Prospective Study
Source: PLoS One. 2012 Oct 31;7(10):e48684. doi: 10.1371/journal.pone.0048684 (PMC3485356; doi:10.1371/journal.pone.0048684)

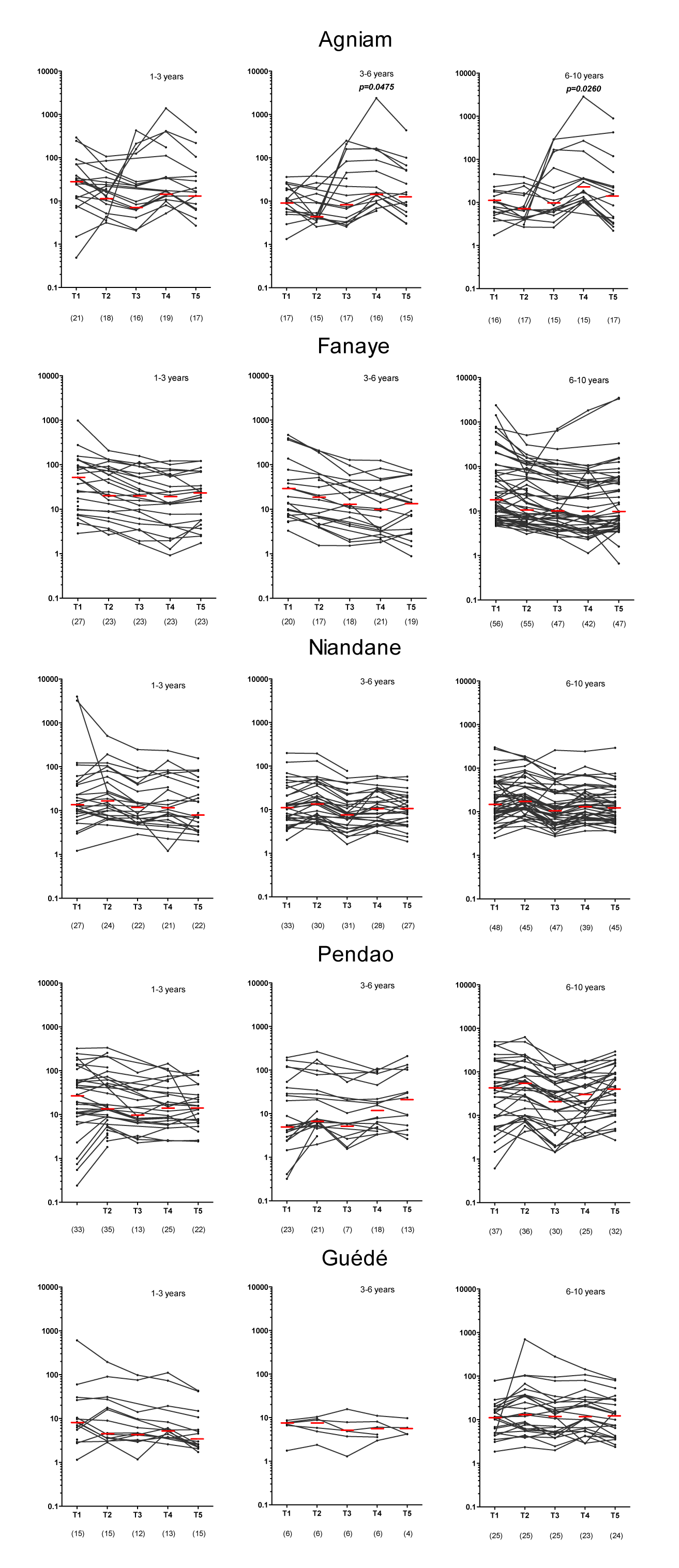

Supplement: Supplementary Material S1 — Individual changes in anti-PT IgG responses over time according to age at first visit in all villages. Different age groups are in separate panels; Data from each individual are connected. Red lines indicate median values at a given visit. The Kruskal-Wallis non-parametric test has been run for each age category in each village. Only significant test results are shown on the graph. (TIF) [file pone.0048684.s001.tif]
